# Training stress, neuromuscular fatigue and well-being in volleyball: a systematic review

**DOI:** 10.1186/s13102-024-00807-7

**Published:** 2024-01-13

**Authors:** André Rebelo, João R. Pereira, Paulo Cunha, Manuel J. Coelho-e-Silva, João Valente-dos-Santos

**Affiliations:** 1 CIDEFES, Centro de Investigação Em Desporto, Educação Física E Exercício E Saúde, Universidade Lusófona, 1749-024 Lisbon, Portugal; 2 COD, Center of Sports Optimization, Sporting Clube de Portugal, 1600-464 Lisbon, Portugal; 3 FCDEF, University of Coimbra, Coimbra, Portugal; 4 CIDAF, University of Coimbra, Coimbra, Portugal

**Keywords:** Team sports, Wellbeing, Health, Performance analysis, Time motion analysis

## Abstract

**Background:**

Volleyball, with its unique calendar structure, presents distinct challenges in training and competition scheduling. Like many team sports, volleyball features an unconventional schedule with brief off-season and pre-season phases, juxtaposed against an extensive in-season phase characterized by a high density of matches and training. This compact calendar necessitates careful management of training loads and recovery periods. The effectiveness of this management is a critical factor, influencing the overall performance and success of volleyball teams. In this review, we explore the associations between training stress measures, fatigue, and well-being assessments within this context, to better inform future research and practice.

**Methods:**

A systematic literature search was conducted in databases including PsycINFO, MEDLINE/PubMed, SPORTDiscus, Web of Science, and Scopus. Inclusion criteria were original research papers published in peer-reviewed journals involving volleyball athletes.

**Results:**

Of the 2535 studies identified, 31 were thoroughly analysed. From these 31 articles, 22 included professional athletes, seven included collegiate-level volleyball athletes, and two included young athletes. Nine studies had female volleyball players, while the remaining 22 had male volleyball athletes.

**Conclusions:**

Internal training load should be collected daily after training sessions and matches with the session rating of perceived exertion method. External training load should also be measured daily according to the methods based on jump height, jump count, and kinetic energy. If force platforms are available, neuromuscular fatigue can be assessed weekly using the FT:CT ratio of a countermovement jump or, in cases where force platforms are not available, the average jump height can also be used. Finally, the Hooper Index has been shown to be a measure of overall wellness, fatigue, stress, muscle soreness, mood, and sleep quality in volleyball when used daily.

**Supplementary Information:**

The online version contains supplementary material available at 10.1186/s13102-024-00807-7.

## Background

Monitoring athletes has become an important and present part of sport preparation. The scientific study of quantifying athletes' training began in the early 1990s with the four methods that were most used at the time: retrospective questionnaires, diaries, physiological monitoring and direct observation [1]. Nowadays, there is a plethora of athletic monitoring methods and technologies, varying from the simplest and cheapest, such as diaries [1], to the most complicated and expensive ones, such as the global positioning system (GPS) [2].

Frequently monitoring the variables related to performance can help coaches to assess the effectiveness of their training programs and update those to better meet the athletes’ needs. Besides, another reason to frequently monitor athletes is to reduce the time lost to illness [3] and injury [4, 5]. By monitoring the weekly training loads, coaches can make better decisions about the changes in the program to ensure that athletes are not exceeding thresholds that put them in higher risk of injury [6] and illness [7]. Furthermore, monitoring the recovery response after a training session or a competitive match can aid practitioners to balance the adaptation process and recovery. This is particularly important to understand the beginning of the period characterized by a decrease in performance in reaction to high loads (i.e., functional overreaching) [8]. Failing to monitor this response can lead to unplanned fatigue followed by a period of inadequate recovery, phenomenon designed by nonfunctional overreaching [9]. This continuum of unplanned fatigue can result in a syndrome defined by overtraining, in which large decrements in performance occur that are associated to psychological disturbances that can last for months [10].

The particularities of the variables mentioned before alongside with the complexity of the majority of team-sports calendar (e.g., short preparation periods and weeks with high volumes of matches and training sessions) can make the training process hard to monitor and prescribe [11]. The management of the balance between training loads and recovery significantly influences a team’s overall fitness, which, in turn, plays a crucial role in their competitive success [4]. One of the team-sports that has a voluminous competitive calendar is professional volleyball. Volleyball is a sport characterized by a diverse range of physical demands, necessitating well-developed energy systems [12, 13]. These include the phosphagen system, which provides immediate energy for high-intensity, short-duration activities like quick sprints or jumps; glycolysis, which predominates in moderate to high-intensity activities lasting from a few seconds up to a minute, contributing to sustained efforts during longer rallies; and the oxidative system, which supports prolonged, lower-intensity activities, crucial for endurance over the course of a match. The effective interplay of these energy systems is essential for optimal performance in volleyball, as players frequently transition between activities of varying intensity and duration [14, 15].

Prior research in the field of volleyball has explored various aspects of athletic performance [12] and recovery [16, 17]. Studies have examined internal and external training loads, investigating how these variables influence players' physiological responses and performance outcomes [18, 19]. Key findings have indicated the importance of monitoring training intensity and volume to optimize player readiness and prevent overtraining [18]. Additionally, research has highlighted the role of neuromuscular fatigue assessments and well-being measures in understanding athletes' responses to training and competition demands [18, 20]. In the realm of these neuromuscular assessments, the vertical jump emerges as a particularly crucial measure in volleyball. This is because the act of jumping is central to key actions such as serving, blocking, and attacking [12]. The vertical jump, therefore, is not just a frequent movement in volleyball but also a critical skill that significantly influences a team's performance and success. It underscores the importance of precisely monitoring and optimizing training loads, as these directly impact an athlete's ability to perform these jumps effectively and consistently. Despite these advancements, there remains a gap in the systematic synthesis of this literature, particularly in integrating these diverse findings to inform monitoring strategies in volleyball. This gap underscores the need for the current systematic review, aiming to consolidate existing knowledge and identify directions for future research.

Moreover, previous research has shown the importance of conducting systematic reviews about training/match monitoring with increasing attention given to the consensus as to which variables related to training load, fatigue, and well-being are most useful [21]. Therefore, the aim of this systematic review was to examine the extent, range, and nature of the evidence on the associations between training load measures, fatigue and well-being assessments used in volleyball training/match monitoring literature to aid the planning of future research.

## Methods

### Registration and protocol

This systematic review was conducted in accordance with the recommendations of the Preferred Reporting Items for Systematic Reviews and Meta-Analyses (PRISMA) 2020 [22]. The study protocol was registered with INPLASY (INPLASY202270059). A PRISMA checklist is provided as a supplementary file (Table S1).

### Eligibility criteria

Inclusion criteria for this systematic review were as follows: (1) original research papers published in peer-reviewed journals in English, French, Spanish, or Portuguese; (2) subjects were volleyball athletes, with no restrictions on age, thereby including youth, collegiate, and adult players; (3) the study involved at least two evaluation points, encompassing a baseline and a post-intervention measurement. The exclusion criteria were: (a) studies not involving human subjects; (b) research not specifically focused on volleyball training or competition; (c) studies lacking empirical data or not presenting clear methodological descriptions. These criteria were designed to ensure an analysis across various age groups and both male and female athletes, providing a holistic understanding of volleyball training and performance.

### Information sources

The literature search was performed from database inception to March 2023 (date when the search was last conducted) in five electronic databases: PsycINFO, MEDLINE/PubMed, SPORTDiscus, Web of Science, and Scopus. The search was developed to consider research articles published online.

### Search strategy

Scientific peer-reviewed published papers written in English, Portuguese, French, and Spanish were eligible for the present systematic review. The search strategy was developed around keywords for Population (volleyball athletes), Exposure (volleyball training or matches), Country (all), and study type (longitudinal). Included terms for the searches were: ‘training load volleyball’, ‘workload volleyball’, ‘rating of perceived exertion volleyball’, ‘RPE volleyball’, ‘well-being volleyball’, ‘wellness volleyball’, ‘fatigue volleyball’, ‘sleep volleyball’, ‘training response volleyball’, ‘neuromuscular fatigue volleyball’, and ‘neuromuscular status volleyball’. The complete search strategy is available in the supplementary file (Table 1).

**Table 1:** Search strategy

Variable	Search terms
Training load	AB OR SU (“training load” OR “training impulse” OR TRIMP OR “external load” OR “internal load” OR duration OR exposure OR RPE OR “rating of perceived exertion” OR summated-heart-rate-zone OR SHRZ OR PlayerLoad OR BodyLoad OR “global positioning system” OR GPS OR accelerometer)
Neuromuscular fatigue	AB OR SU (“neuromuscular fatigue” OR “neuromuscular function” OR “neuromuscular performance” OR “neuromuscular power” OR fatigue OR fatiguing OR fatigability)
Well-being	AB OR SU (wellbeing OR well-being OR “well being” OR wellness OR health OR psychological OR “mental state*” OR “state of mind” OR affect OR affective OR affects OR mood* OR emotion* OR anxiety OR confidence OR self-esteem OR self-efficacy OR motivation OR depression OR stress OR tension OR feeling* OR “physical state” OR “physical functioning” OR “perceived recovery” OR “perceived strength” OR soreness OR “quality of life” OR readiness OR vitality OR vigor OR vigour OR sleepiness OR “sleep quality” OR fatigue OR tiredness OR alertness OR distress OR “social function” OR appetite OR overtrain* OR overreach*)
Volleyball	AB OR SU (volleyballer* OR “volleyball player*” OR “volleyball athlete*”)
Final search	training load OR neuromuscular fatigue OR well-being AND volleyball

*AB* abstract, *SU* subject, * truncation, “” phrase search

### Selection and data collection process

All retrieved papers were exported to CADIMA software, a tool designed to increase the efficiency of the evidence synthesis process and facilitate reporting of all activities to maximize methodological rigor [23]. Duplicates were automatically removed. Titles and abstracts of potentially relevant papers were screened by two reviewers (A.R. and J.R.P.). Disagreements between authors were solved through discussion and, when necessary, the remaining authors (P.C., M.J.C-S. and J.V-S.) were involved. Full‐text copies were acquired for all papers that met title and abstract screening criteria. Full‐text screening was performed by two reviewers (A.R. and J.R.P.). Again, any discrepancies were discussed until the authors reached an agreement and consulted the four other authors when required. In the process of article selection, inter-rater reliability was quantitatively assessed using the Cohen kappa coefficient. For the initial title and abstract screening, the kappa coefficient was 0.810. Similarly, for the full-text review phase, the kappa coefficient was 0.979.

### Data extraction

Data were extracted from each article by the lead author (A.R.). Data not provided or presented non-numerically were identified as “not reported”. The following data, when possible, were extracted from each article: (1) participants’ characteristics (sample size, sex and age); (2) participants’ level (young, collegiate or professional); (3) monitoring period (i.e., seasonal phase(s) and duration); (4) training load measures (e.g., RPE, heart rate, time motion analysis); (5) neuromuscular fatigue tests (e.g., heart rate, biochemical markers); (6) well-being assessment methods (e.g., scale, questionnaire).

### Risk of bias assessment

Methodological quality was assessed using a modified version of the Downs and Black [24] checklist for assessing the methodological quality of randomized and nonrandomized healthcare interventions. This checklist has been validated for use with observational study designs [24] and has been previously used to assess methodological quality in systematic reviews assessing cross-sectional and longitudinal studies [25, 26]. The number of items from the original checklist can be tailored to the scope and needs of the systematic review, with 10–15 items used in previous systematic reviews [25, 26]. For this review, 11 items in the checklist were deemed relevant (Table S3). Each item is scored as “1” (yes) or “0” (no/unable to determine), and the scores for each of the 11 items are summed to provide the total quality score. The quality of each included article was rated against the checklist independently by two authors (A.R. and J.R.P.). Any disparity in the outcome of the quality appraisal was discussed, and a third author (J.V-S.) was consulted if a decision could not be reached. In the assessment of methodological quality and risk of bias, inter-rater reliability was quantitatively evaluated using the Cohen kappa coefficient. The kappa value obtained was 0.903.

### Data synthesis

Results were not pooled as the studies were heterogeneous in their methods, data, and context. Instead, we presented a narrative synthesis of the findings from included studies. We identified three categories of monitoring interventions through the process of reviewing the included studies. The definitions of these interventions are provided in the supplementary file (Table S2). Summary tables were provided as means and standard deviations were reported for age of participants, body mass, and body height. The period of each study (i.e., pre-season, competitive period, or both) and the duration of the study, in weeks, were also reported.

## Results

### Study selection

**Fig.1:**
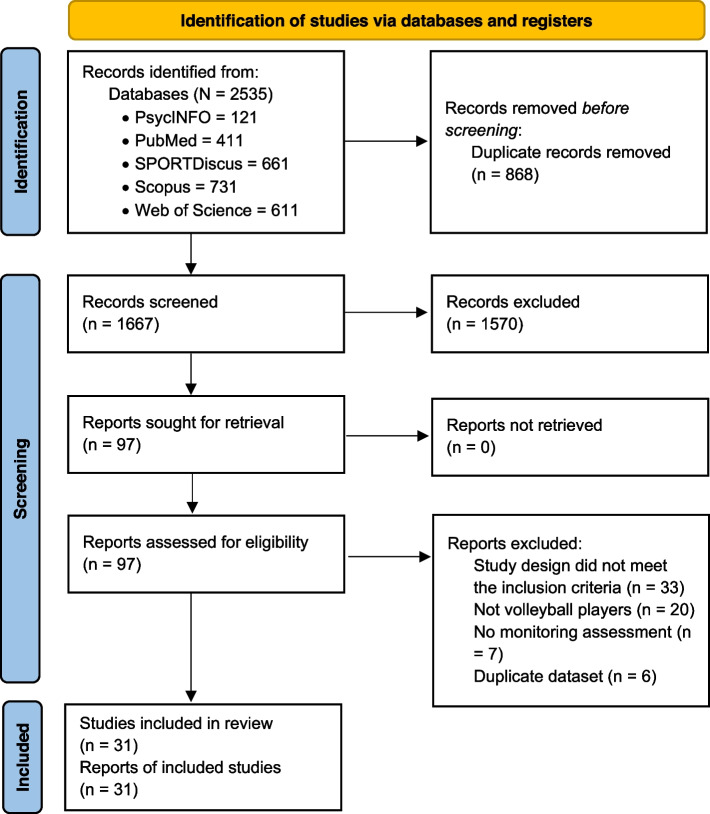
PRISMA flow diagram

The electronic search yielded 2535 articles (PsycINFO = 121, PubMed = 411, SPORTDiscus = 661, Scopus = 731, Web of Science = 611). A total of 868 duplicate records were removed, and a further 1570 irrelevant articles were excluded based on title and abstract; 97 fulltext articles were screened and 66 were removed, leaving 31 articles for inclusion in the review. Reasons for exclusion were study designs did not meet the inclusion criteria (*n* = 33), no volleyball players in the sample (*n* = 20), failure to perform any monitoring strategy (*n* = 7), and duplicate dataset (*n* = 6). The full results of the search are presented in Fig. 1.

### Risk of bias in studies

The ratings from the quality appraisal for each article are presented in the supplementary file (Table S4). Methodological quality scores ranged from 7 to 9 out of 11. The predominant concerns identified in the evaluation of these studies centre around issues of external validity, particularly the representativeness of the study participants. This limitation significantly hampers the generalizability of the findings. The studies fall short in ensuring that the subjects included are reflective of the broader population from which they are drawn, raising questions about the applicability of their conclusions beyond the specific sample studied. In line with previous literature using the Downs and Black checklist [25, 26], no articles were excluded based on methodological quality.

### Study characteristics

Study characteristics for all 31 included studies are presented [16–18, 27–54] (Table 2). From these 31 articles, 22 included professional athletes [16–18, 28, 30–32, 34–36, 38–43, 46, 48, 50–53], seven were collegiate-level volleyball athletes [27, 29, 33, 37, 44, 45, 47], and two included young athletes [49, 54]. Nine articles used female volleyball players [27–29, 32, 33, 37, 44, 45, 47], while the remaining 22 were male volleyball athletes [16–18, 30, 31, 34–36, 38–43, 46, 48–54].

**Table 2:** Characteristics of the included studies

Author, year	N	Level	M/F	Age (years)	Duration
Rebelo et al., 2023 [18]	15	Professional	M	28.51 ± 5.39	5 weeks
Herring and Fukuda, 2022 [27]	9 (5 across both seasons)	Collegiate	F	NR	2 seasons
Berriel et al., 2022 [30]	16	Professional	M	23.60 ± 4.93	Pre-season
Rabbani et al., 2021 [28]	13	Professional	F	25.8 ± 3.0	4 training camps (≈1 month)
Haraldsdottir et al., 2021 [29]	17	Collegiate	F	19.6 ± 1	1 season
Andrade et al., 2021 [31]	15	Professional	M	24 ± 4	1 season
Timoteo et al., 2021 [17]	14	Professional	M	26.7 ± 5.5	1 season
Ungureanu et al., 2021 [32]	12	Professional	F	22 ± 4	1 season
Kupperman et al., 2021 [33]	11	Collegiate	F	19.36 ± 1.27	1 season
Berriel et al., 2020 [34]	13	Professional	M	23.80 ± 5.40	Pre-season
Horta et al., 2020 [35]	9	Professional	M	26.4 ± 4.0	1 season
García-de-Alcaraz et al., 2020 [36]	11	Professional	M	28.0 ± 6.12	1 season
Roy et al., 2020 [37]	15	Collegiate	F	NR	1/2 season
Clemente et al., 2020 [38]	13	Professional	M	31.0 ± 5.0	1 season
Lima et al., 2020 [39]	8	Professional	M	23.0 ± 5.22	15 weeks
Duarte et al., 2019 [40]	14	Professional	M	24.0 ± 3.59	1 season
Clemente et al., 2019 [41]	13	Professional	M	31.0 ± 5.0	1 season
Horta et al., 2019 [42]	12	Professional	M	26.9 ± 4.6	Pre-season
Silva et al., 2019 [43]	8	Professional	M	23.0 ± 0.2	1 season
Roy et al., 2019 [44]	15	Collegiate	F	NR	1/2 season
Hyatt and Kavazis, 2019 [45]	8	Collegiate	F	NR	1 season
Skazalski et al., 2018 [46]	14	Professional	M	NR	1 season
Castello et al., 2018 [47]	10	Collegiate	F	19.80 ± 1.23	8 weeks
Mendes et al., 2018 [48]	13	Professional	M	31 ± 5.0	1 season
Tavares et al., 2018 [49]	13	Young	M	18 ± 1	1 week
Debien et al., 2018 [50]	15	Professional	M	24.0 ± 3.6	1 season
Brandão et al., 2018 [51]	14	Professional	M	26.7 ± 5.5	1 season
Nogueira et al., 2017 [52]	12	Professional	M	23.50 ± 3.39	1 season
Horta et al., 2017 [53]	15	Professional	M	G1: 25.9 ± 3.8 G2: 23.1 ± 3.1	10 weeks (without matches)
Timoteo et al., 2017 [16]	12	Professional	M	26.7 ± 5.5	1 week (5 matches)
de Freitas et al., 2015 [54]	7	Young	M	15.8 ± 0.5	Pre-season

*F* female,  *M* male, *NR* not reported, *G1* group 1, *G2* group 2

### Quantifying training stress in volleyball athletes

Quantifying training stress can be done in different ways. The most common one can be achieved by multiplying the training session intensity by the training session duration. Training load can be either internal or external [55]. Internal training load refers to the physiological stress that a training session induces in the athlete [55]. Measures such as heart rate (HR) and rating of perceived exertion (RPE) are the most common methods to monitor internal load [2]. On other hand, external training load is defined as the physical work prescribed in the training plan [55]. The most common method of monitoring external load is with time-motion analysis devices, such as GPS, accelerometers, or inertial motion units (IMUs) [2].

The effects of different training loads measurements have been investigated in volleyball with durations ranging from one week [16, 49] to two seasons [27] (Table 3). Moreover, the effects of single training load measurement (i.e., internal, or external) [16, 17, 27, 28, 30–32, 35–38, 40–42, 44, 46–51, 53, 54] or a combination of both training load measurements [18, 33, 39, 43] have been investigated. The session rating of perceived exertion (sRPE) (77%) [16–18, 28, 30–33, 35, 37–44, 47–51, 53, 54] and the IMUs (16%) [18, 27, 39, 43, 46] are the most commonly used training load measurement strategies in volleyball. Other training load measures investigated in the volleyball literature include HR [32], accelerometers [33], and video-cameras [36].

**Table 3:** Training stress monitoring strategies and protocols in the volleyball literature

Author, year	Training stress measurements	Methodology	Results
Rebelo et al., 2023 [18]	Internal load—sRPE External load—IMU (jump metrics)	Jumping metrics and sRPE of each training session	wITL (range): 1229.00 ± 247.74 to 2188.13 ± 693.36 wETL (range): 11,144.92 ± 3648.12 kJ to 18,328.99 ± 8358.20 kJ
Herring and Fukuda, 2022 [27]	External load—IMU (jump metrics)	53 matches across 2 seasons	MB—HT: 47.4 ± 5.4; OJC: 89.2 ± 30.7; OJR = 0.95 ± 0.21 OH—HT: 51.9 ± 2.2; OJC: 72.8 ± 22.8; OJR = 0.77 ± 0.13 RSH—HT: 45.4 ± 11.4; OJC: 50.3 ± 22.1; OJR = 0.57 ± 0.19
Berriel et al., 2022 [30]	Internal load—sRPE	sRPE of each training session	wITL (range): 1388 ± 111 to 3852 ± 149
Rabbani et al., 2021 [28]	Internal load—sRPE	sRPE of each training session	sRPE (range): 1052 ± 163 to 1105 ± 121
Andrade et al., 2021 [31]	Internal load—sRPE	sRPE of each training session	PS—TWTL: 3,512.84 ± 876.48 CPI—TWTL: 2,843.93 ± 1,026.14 CPII—TWTL: 2,696.40 ± 933.51
Timoteo et al., 2021 [17]	Internal load—sRPE	sRPE of each training session	PS—TWTL: 3,492.75 ± 2,320.68 CP—TWTL: 3,207.02 ± 2,423.04
Ungureanu et al., 2021 [32]	Internal load—sRPE, HR	HR and sRPE of each training session	MB—sRPE: 534; EHR: 207 OH—sRPE: 402; EHR: 172 RSH—sRPE: 463; EHR: 206 L—sRPE: 313; EHR: 180 SE—sRPE: 351; EHR: 233
Kupperman et al., 2021 [33]	Internal load—sRPE External load—accelerometer (jump metrics, COD, accelerations)	sRPE and accelerometer in each training session and game	Training sessions: OJC: 90.9 ± 51.2; COD: 247.5 ± 121.7; ACC: 93.6 ± 46.9; DEC: 94.8 ± 52.9 Games: OJC: 81.1 ± 49.8; COD: 229.4 ± 124.8; ACC: 85.2 ± 47.2; DEC: 66.0 ± 39.7
Horta et al., 2020 [35]	Internal load—sRPE	sRPE of each training session	PS—TWTL: 3,228.44 ± 521.96 CPI—TWTL: 3,369.44 ± 605.33 CPII—TWTL: 2,973.22 ± 727.23
García-de-Alcaraz et al., 2020 [36]	External load—camera (jump count)	each training session	MB—OJC: 41,432 OH—OJC: 40,694 RSH—OJC: 22,997 SE—OJC: 13,226
Roy et al., 2020 [37]	Internal load—sRPE	sRPE of each training session and game	sRPE: 566 ± 260
Clemente et al., 2020 [38]	Internal load—sRPE	sRPE of each training session and game	CPI—ACWR: 1.10 ± 0.13; M: 4.28 ± 1.23 CPII—ACWR: 1.66 ± 0.15; M: 3.39 ± 0.69
Lima et al., 2020 [39]	Internal load—sRPE External load—IMU (jump metrics)	Jumping metrics and sRPE of each training session	sRPE and OJC was higher in MD-2 and MD-3 than in MD-1
Duarte et al., 2019 [40]	Internal load—sRPE	sRPE of each training session and game	CPI—TWTL: 4,546.0 ± 620.9 CPII—TWTL: 4,006.6 ± 687.6
Clemente et al., 2019 [41]	Internal load—sRPE	sRPE of each training session and game	CPI > TWTL > CPII
Horta et al., 2019 [42]	Internal load—sRPE	sRPE of each training session	The TWTL increased progressively from Week 2 to Week 6
Silva et al., 2019 [43]	Internal load—sRPE External load—IMU (jump metrics)	Jumping metrics and sRPE of each training session	sRPE: MD-1: 462.04 ± 330.05; MD-2: 586.68 ± 365.66; MD-3: 477.44 ± 267.34; MD-4: 466.68 ± 295.71; MD-5: 430.21 ± 215.77 OJC: MD-1: 106.40 ± 42.77; MD-2: 143.10 ± 60.11; MD-3: 120.31 ± 46.58; MD-4: 118.87 ± 68.61; MD-5: 106.56 ± 35.65
Roy et al., 2019 [44]	Internal load—sRPE	sRPE of each training session	sRPE: 566 ± 260
Skazalski et al., 2018 [46]	External load—IMU (jump metrics)	Jumping metrics each training session and game	MB—OJC: 92 OH—OJC: 62 RSH—OJC: 75 SE—OJC: 121
Castello et al., 2018 [47]	Internal load—sRPE	sRPE of each training session	wITL: 2484.32
Mendes et al., 2018 [48]	Internal load—sRPE	sRPE of each training session	Preparatory weeks: training load had an undulating distribution during the week; regular and congested weeks: training load was higher at the beginning of the week
Tavares et al., 2018 [49]	Internal load—sRPE	sRPE of each training session	sRPE had an undulating distribution during the week
Debien et al., 2018 [50]	Internal load—sRPE	sRPE of each training session and game	PS—TWTL: 3,748 ± 472 CPI—TWTL: 2,858 ± 472 CPII—TWTL: 3,728 ± 650
Brandão et al., 2018 [51]	Internal load—sRPE	sRPE of each training session	Preparatory weeks: training load had an undulating distribution during the week; regular and congested weeks: training load was higher at the beginning of the week
Horta et al., 2017 [53]	Internal load—sRPE	sRPE of each training session	First team players > TWTL > reserve players
Timoteo et al., 2017 [16]	Internal load—sRPE	sRPE of each training session and game	sRPE: Day 2 > Day 1 > Day 6 > Day 3 > Day 5 > Day 4
de Freitas et al., 2015 [54]	Internal load—sRPE	sRPE of each training session	Week 1—TWTL: 1,922 ± 654 Week 2—TWTL: 1,530 ± 691 Week 3—TWTL: 1,874 ± 528 Week 4—TWTL: 1,568 ± 312

*COD* change of direction, *CP* competitive period, *CPI* competitive period I, *CPII* competitive period II, *HER* Edwards Heart Rate, *HT* mean jump height from all jumps (cm), *IMU* inertial motion unit; *L* libero, *M* monotony, *MB* middle blocker, *OH* outside hitter, *OJC* overall jump count, *OJR* overall jump rate (jumps/min), *PS* pre-season, *RSH* right-side hitter, *SE* setter, *TWTL*  total weekly training load (arbitrary units), *sRPE* session rating of perceived exertion, *wITL*  weekly internal training load (arbitrary units)

### Quantifying fitness and fatigue in volleyball athletes

The reduction in maximal voluntary contractile force is designated by neuromuscular fatigue and tests to detect this type of fatigue are broadly used in sport [2]. Low-frequency fatigue (i.e., resulted from high-force, high-intensity, or repeated stretch–shortening cycles muscle actions) is frequently a topic of interest while monitoring athletes [56]. Consequently, many research studies have established the reliability and validity of vertical jumps as an indicator of neuromuscular fatigue in athletes [57]. One of the most valid measures of fatigue is the ratio of flight time to contraction time (FT:CT), which can be explained by the fact that time-related variables are more sensitive to fatigue [58]. Nevertheless, other measures such as jump height, peak and mean power, and peak force are also popular among coaches [59].

In addition to being used to monitor training stress, submaximal exercise protocols and physiological markers such as HR can be used as objective markers of fatigue. Heart rate variability (HRV) is widely used, in particular the natural logarithm of the square root of the mean sum of squared differences between adjacent normal RR intervals (Ln rMSSD) [60]. Another monitoring tool that can be used is the recovery period after a training session, indicated with the heart rate recovery (HRR) [61]. Finally, examining hormonal and biochemical markers can provide a good indicator of athletes’ adaptation process [62].

Only five studies included fitness and fatigue measurements as tools to monitor volleyball athletes [18, 28, 34, 42, 49] (Table 4). The countermovement jump (CMJ) is the most used fatigue measurement strategy in volleyball [18, 28, 42, 49]. Other fitness and fatigue monitoring tools are hormonal and biochemical markers [34, 42] and HR variables [28].

**Table 4:** Fitness and fatigue monitoring strategies and protocols in the volleyball literature

Author, year	Fitness and fatigue measurements	Design	Results
Rebelo et al., 2023 [18]	Fatigue—CMJ	3 maximal attempts of the CMJ on Matchday -1	CMJ (range): 44.46 ± 6.09 to 47.24 ± 7.21
Rabbani et al., 2021 [28]	Fatigue—CMJ, HR	3 maximal attempts of the CMJ and a submaximal running test at the beginning of the first training session for each camp; Ln rMSSD after waking up (supine and seated)	CMJ (range): 32.1 ± 3.5 to 35.1 ± 4.1 HRex (range): 148.0 ± 8.6 to 156.8 ± 7.6 HRR (range): 37.9 ± 9.8 to 41.7 ± 15.3
Berriel et al., 2020 [34]	Biochemical markers—CK	6 times in different weeks of the 16 studied	CK increased after the first weeks of training and remained stable until the beginning of the pre-competitive period, at which time they dropped significantly
Horta et al., 2019 [42]	Fatigue—CMJ Biochemical markers—CK, T, Cr	CMJ and blood samples 4 times each 14 days	CMJ: M1: 46.92 ± 5.75; M2: 45.55 ± 6.16; M3: 46.91 ± 5.95; M4: 46.94 ± 5.92 T: M1: 511 ± 100; M2: 559 ± 122; M3: 487 ± 117; M4: 549 ± 61 Cr: M1: 17.3 ± 7.1; M2: 15.5 ± 6.1; M3: 14.2 ± 3.6; M4: 13.8 ± 3.8
Tavares et al., 2018 [49]	Fatigue—CMJ	Days 1, 2, 4 and 5	CMJ height decreased during the week

*CK*   creatine kinase, *CMJ* countermovement jump (cm), *Cr* cortisol (ng.dL-1), *HRR* heart rate recovery (b/min), *HRex* submaximal exercise heart rate (b/min), *Ln rMSSD* natural logarithm of the square root of the mean sum of squared differences between adjacent normal RR intervals, *M1* moment one, *M2* moment two, *M3* moment three, *M4* moment four, *T* testosterone (ng.dL-1)

### Quantifying well-being in volleyball athletes

Questionnaires can be useful to monitor athletes’ levels of stress [1] and identify those at greater risk of becoming injured [63]. Research has shown that athletes often have a mood disturbance while developing symptoms of overreaching and overtraining [2]. Therefore, assessing athlete’s mood state and level of tension through tools such as the Profile of Mood States (POMS) and the Brunel Mood Scale (BRUMS) can be useful [64]. Wellness inventories, like the Hooper index [65], are also common if the goal is to gather as much information as possible about different metrics, such as fatigue, stress, sleep, or recovery.

The current literature search returned 22 studies that applied some form of well-being questionnaire [16–18, 28, 29, 31–35, 38, 40–42, 44, 45, 48–52, 54] (Table 5). The Hooper index [16, 28, 32, 38, 41, 44, 48], the Total Quality Recovery (TQR) scale [16, 17, 31, 35, 40, 50, 51], and general wellness questionnaires [18, 29, 33, 40, 49, 51, 52] are the most commonly used well-being measurement strategies in volleyball. Other well-being measuring tools investigated in the volleyball literature include the Recovery Stress Questionnaire for Athletes (RESTQ-Sport) [34, 42, 54] and the POMS [35].

**Table 5:** Well-being monitoring strategies and protocols in the volleyball literature

Author, year	Well-being measurements	Design	Results
Rebelo et al., 2023 [18]	Well-being—Questionnaire	Daily before the first training session	No differences observed in most wellness items measured during the 5 weeks
Rabbani et al., 2021 [28]	Well-being—Hooper's index	Daily before the first training session	2.19 ± 0.35 to 2.24 ± 0.30
Haraldsdottir et al., 2021 [29]	Well-being—Questionnaire	Daily before the first training session	7.9 ± 1.2
Andrade et al., 2021 [31]	Recovery—TQR Scale	Daily before the first training session	PS—TQR: 14.27 ± 1.50 CPI—TQR: 15.26 ± 1.43 CPII—TQR: 15.06 ± 1.47
Timoteo et al., 2021 [17]	Recovery—TQR Scale	Once per week	No injured players—16.67 ± 6.09 Injured players (overuse)—15.26 ± 2.66 Injured players (trauma)—14.63 ± 2.20
Ungureanu et al., 2021 [32]	Well-being—Hooper's index	Daily before the first training session	MB—15.9 OH—13.8 RSH—15.3 L—15.6 SE—15.0
Kupperman et al., 2021 [33]	Well-being—Questionnaire	Daily before the first training session or game	F: 2.1 ± 0.9 M: 1.7 ± 0.9 S: 2.1 ± 1.1 SO: 1.9 ± 0.9
Berriel et al., 2020 [34]	Well-being—RESTQ-Sport	6 times in different weeks of the 16 studied	M1: 169.01 ± 94.42; M2: 673.92 ± 461.45; M3: 520.77 ± 348.87; M4: 631.76 ± 579.30; M5: 270.78 ± 245.37; M6: 330.23 ± 206.98
Horta et al., 2020 [35]	Well-being—POMS Recovery—TQR Scale	Daily before the first training session or game	PS—V: 20.62 ± 3.96; F: 11.82 ± 2.76; TQR: 14.95 ± 0.79 CPI—V: 18.31 ± 4.62; F: 12.89 ± 2.73; TQR: 15.33 ± 0.94 CPII—V: 18.76 ± 3.74; F: 8.65 ± 2.65; TQR: 15.74 ± 1.01
Clemente et al., 2020 [38]	Well-being—Hooper's index	Daily before the first training session or game	CPI—Weekly index: 61.82 ± 11.57 CPII—Weekly index: 54.46 ± 16.68
Duarte et al., 2019 [40]	Well-being—Questionnaire Recovery—TQR Scale	First and last training/game of the week	CPI—TQR: 16.7 ± 1.1 CPII—TQR: 15.9 ± 1.1
Clemente et al., 2019 [41]	Well-being—Hooper's index	Daily before the first training session or game	CPI > index > CPII
Horta et al., 2019 [42]	Well-being—RESTQ-Sport	Daily before the first training session	The General Well-being value was lower at Weeks 2 and 6 than at baseline. The Injury at Week 4 was larger than that at baseline
Roy et al., 2019 [44]	Well-being—Hooper's index	Daily before the first training session	10.3 ± 3.5
Hyatt and Kavazis, 2019 [45]	Stress scale	7 times in different parts of the season	Perceived stress peaked during the mid-season
Mendes et al., 2018 [48]	Well-being—Hooper's index	Daily before the first training session	Regular weeks: best index score MD; congested weeks: index disrupted several days of the week
Tavares et al., 2018 [49]	Well-being—Questionnaire Muscle soreness—Questionnaire	Daily before the first training session	Decrease in wellness scores and increase in fatigue and soreness during the week
Debien et al., 2018 [50]	Recovery—TQR Scale	Daily before the first training session	PS—TQR: 15.63 ± 0.80 CPI—TQR: 15.02 ± 1.03 CPII—TQR: 14.75 ± 0.79
Brandão et al., 2018 [51]	Well-being—Questionnaire Recovery—TQR Scale	Daily before the first training session	Regular weeks—TQR: 15.61 ± 0.33 Congested weeks—TQR: 15.58 ± 0.57
Nogueira et al., 2017 [52]	Well-being—Questionnaire	First and last training of the week	Decrease in wellness scores and increase in fatigue and soreness during the week
Timoteo et al., 2017 [16]	Well-being—Hooper's index Recovery—TQR Scale	Daily before the first training session or game	Hooper: Day 1 > Day 2 > Day 5 > Day 3 > Day 6 > Day 4 TQR: Day 1 > Day 2 > Day 6 > Day 5 > Day 4 > Day 3
de Freitas et al., 2015 [54]	Well-being—RESTQ-Sport	Daily before the first training session	Higher fatigue and injury scores during the pre-season period

All data are in arbitrary units

*CPI* competitive period I, *CPII* competitive period II, *F* fatigue, *M* mood, *PS* pre-season, *RESTQ-Sport* Recovery Stress Questionnaire for Athletes, *S* stress, *SD* sleep duration, *SO*   soreness, *SQ* sleep quality, *TQR* total quality recovery scale, *V* vigor

## Discussions

**Fig.2:**
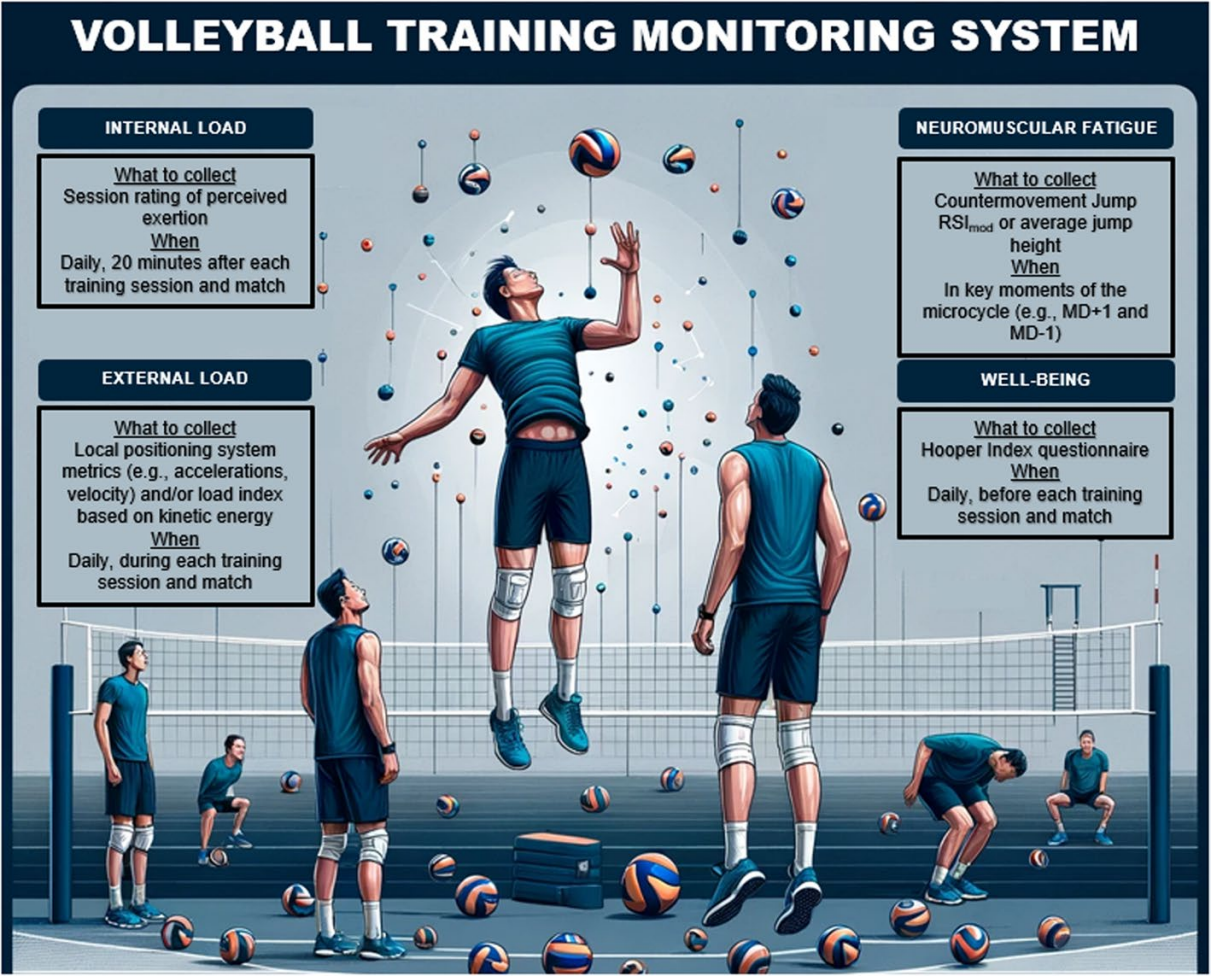
General recommendations for assessing the training load, neuromuscular fatigue, and well-being of volleyball players

Literature that has evaluated the effect of all monitoring strategies (i.e., training stress, fitness and fatigue, and well-being) during volleyball training and/or competition is limited. Besides, there is a small number of studies describing the external training load when compared with the internal training load. Furthermore, not only fitness and fatigue monitoring studies are limited, but also have questionable methodologies within volleyball athletes. A sample monitoring system for volleyball is suggested in Fig. 2.

### Training stress in volleyball

Seven studies analysed the internal load of volleyball players during the pre-season with the sRPE [17, 30, 31, 41, 42, 50, 54]. During the first weeks of pre-season the internal load of the players is defined by a progressive increase characterized by a decrease in performance [30, 31, 42]. This can also be seen with external training load measures, as jump load is higher during the first phase of pre-season [36]. To better prepare athletes for the start of the competition phase, this periodization approach is common in team-sports during the pre-season [54, 66]. Coaches are advised to introduce the load progressively and, in the middle of the pre-season period, decrease the training loads to allow recovery and better balance the fitness-fatigue relationship [67]. In fact, elevated injury rates have been observed during this period in other sports [68]. This is in line with what is reported in volleyball’s literature, as weekly workloads, acute-chronic workload ratio (ACWR), and incidence of injury values are higher during the pre-season period [17, 50]. Coaches and practitioners should evaluate athletes’ fitness in the beginning of the pre-season period and assess what were the workloads that players were familiarized during the off-season so that weekly internal training load peaks do not occur.

Sixteen studies analysed the internal training load of volleyball athletes during the competitive period with the sRPE method [17, 18, 31–33, 35, 37–41, 43, 44, 48, 50, 51]. It can be observed that volleyball periodization is characterized by a wave distribution of the training load during this period [31, 35, 37, 40, 48, 50]. This is distinctive of sports in which the pre-season period is short compared to the competitive period with the objective to adapt the stress applied during training sessions [11]. Due to various travels made and games played against teams of different levels the number of training sessions reduce during the competitive period [66]. Therefore, this wave distribution of the training load can avoid a possible decrement in performance. This can be done by increasing training loads in weeks in which the team has a low possibility of winning or losing the game [11, 66]. In a more in-depth analysis, results of the literature indicate that during the first phase of the competitive period, volleyball athletes experience higher internal loads compared to the second phase of the same period [31, 35, 38, 40, 41]. The first phase of the competitive period of volleyball professional season is characterized by a focus on the development of fitness components while the second phase comprises the most specific training sessions (technical and tactical skills) [11]. Thus, this can explain these differences in internal load levels observed during the competitive period. Moreover, while looking into a single week, it can be observed that higher sRPE values are recorded during the middle of the week and lower values at the end of the week [39, 43, 48]. This is a common strategy to optimize the adaptation process in team sports by augmenting athletes’ recovery status by reducing training loads [11].

There are significant differences in competition and in training jump count, jump height and jump load between positions in female [27] and male volleyball athletes [33, 36, 46]. Outside hitters had the highest jump height followed by middle blockers and right-side hitters [27]. Female [27] and male [36, 46] volleyball middle blockers showed a higher jump count and jump rate compared to outside hitters and right-side hitters. This is in line with another study with female volleyball athletes that reported that middle blockers experienced both a higher HR-method internal training load and sRPE than the rest of the players [32]. Middle blockers are often required to be involved in every defensive blocking aspect of the game [69], hence their higher values of both external and internal training load. Nevertheless, HR measures of internal training load should be interpreted with caution. While the HR represents a valid means through which to measure exercise intensity in endurance sports, these methods are questionable in team sports, such as volleyball, which are characterized by short but maximal anaerobic efforts [70]. In fact, the results of one study stated no association between well-being and HR-based internal training load [32]. Thus, given the limitations inherent in using the HR for monitoring the intensity of volleyball training sessions, coaches are advised to not use HR-based methods to quantify training stress in this sport.

### Fitness and fatigue in volleyball

One study demonstrated that submaximal exercise heart rate (HRex) values decreased over a period of 4 weeks [28]. Reductions in HRex are generally associated with improved aerobic fitness, while elevations in HRex are related to acute fatigue or loss of fitness [71]. One study also showed positive associations between seated Ln rMSSD and training load (i.e., sRPE) in female volleyball athletes [28]. These results must be interpreted carefully as these positive associations can vary depending on how loads are being tolerated by athletes. If training loads increase in response to increments in fitness and performance, then seated Ln rMSSD will reduce [72]. On other hand, if converse cardiac-autonomic responses are stimulated through mechanisms of fatigue resulted from high training loads, then seated Ln rMSSD will increase [73]. These inconsistencies in associations between Ln rMSSD and training load show the importance of monitor various markers of fatigue, fitness, load, and well-being. Previous research showed that HRV values return to baseline 24 h after an intense exercise bout in the supine position [74]. Therefore, it can be hypothesized that high training loads induces greater fluctuations in the seated Ln rMSSD compared to supine Ln rMSSD. Thus, coaches and practitioners should have this into consideration when monitoring fatigue of volleyball athletes through HRV.

In response to a high-load exercise, various enzymes and blood markers, such as creatine kinase (CK), increase [63]. This type of exercises induces muscle damage and since CK is released from muscle cells to blood, practitioners have been using CK levels to assess the degree of muscle damage [75]. According to the search conducted, volleyball athletes experience an increase of CK levels during the first weeks of pre-season and a decrease in the final weeks [34, 42]. This is in line with what was already mentioned in this manuscript about the levels of sRPE during the pre-season period. It is expected to observe higher increment in CK levels in individuals with lower physical fitness [75], particularly during initial training periods (i.e., pre-season) characterized as an initial training time followed by a period with no structured training. This also indicates that CK levels increase in response to high training loads, which is in line with what was previously reported [75]. However, CK has a large variability [76] and personnel involved in the collection of this marker must understand the importance of establishing baseline values from many samples over several days. Testosterone and cortisol are other two markers that are associated with cellular catabolism, anabolism, and overreaching [62]. Literature shows that during volleyball pre-season, both testosterone and cortisol levels do not change [42]. This is probably an indicator that volleyball pre-season is not enough to induce disturbances in the balance of the immune system.

Results from a study conducted during the pre-season showed that the CMJ height did not change during a 6-week period, assessed four times during this time-window [42]. Another study revealed that, across a single training week, the CMJ jump height decreased [49]. Both studies’ methodologies indicated that the best of all jumps was retained for analysis. However, when the comparison between highest and average results is possible, the averaged jump results is more sensitive than the highest jump in detecting fatigue or supercompensation effects [77]. Therefore, these results should be interpreted with caution and volleyball coaches should have into consideration that averaged CMJ performance without arm swing should be used to track neuromuscular status.

### Well-being in volleyball

One study reported well-being measures, such as mood, soreness, and sleep duration, as independent predictors of injury in female volleyball athletes [29]. This is aligned with other non-volleyball studies [78]. According to the literature, athletes do not get the sleep duration that is recommended [79] which is a minimum of 7 h to minimize injury risk [80]. Therefore, volleyball staff should seek to include these subjective markers into their daily training monitoring routines to identify athletes with higher injury risk.

Volleyball athletes’ recovery state is lower in the final stage of the pre-season, compared to other points of the competitive period [31]. In the last phase of the pre-season, coaches are advised to employ a taper strategy to avoid the undesirable outcomes of fatigue already mentioned in the beginning of the present manuscript, like nonfunctional overreaching [8]. In fact, the results of a study with professional male volleyball players showed that the odds of injury were inversely proportional to the values of TQR scale (i.e., the less recovered the player, the greater the odds of sustaining an injury) [17]. Likewise, athletes’ readiness to start the competitive period is important since the perception of stress increase whereas their perception of recovery decrease during a volleyball pre-season [31, 34, 54]. The results from other studies suggested that the RESTQ-Sport [42] and the Hooper index [16, 44] are sensitive to an increase in the training load in volleyball athletes, showing promising results as tools to indicate early symptoms of overtraining. Consequently, balancing pre-season training stress and recovery is essential so athletes’ adaptation process is optimized for match-days.

During periods of congested travels and games, volleyball athletes reported poorer well-being responses in questionnaires [16, 33, 35, 40, 48, 51, 52]. Time lost to travel, and the ensuing disruption of routines and training schedules may inhibit the use of recovery and medical interventions. Since travels can decrease the well-being and increase athletes’ risk for illness, coaches and staff should implement some strategies, such as: provide adequate recovery time after travels; avoid flying on the same day as match-day; and encourage athletes to drink water during travels [81]. By tracking well-being values coaches can make informed decisions about the demands that incur from both in and out of sport activities.

During the last stage of the competitive period, higher levels of stress can be observed in professional volleyball athletes [38, 41]. Anxiety of a pre-match situation seems to impact the perception of stress levels by professional athletes [82]. This stage is characterized by the decisive matches of the season. On other hand, stress levels in collegiate volleyball athletes may not be as heavily influenced by athletic events during the season and may be more a consequence of the temporal relation to the academic school year [45]. Therefore, challenges that occur in social and academic settings are the offset to higher stress levels in collegiate athletes.

### Limitations, strengths, and recommendations for future research

Many conclusions can be drawn from the available literature on the monitorization strategies in the volleyball context. Studies addressing the responses of the three types of monitorization strategies in volleyball are limited [18, 28, 42, 49]. Of these four studies, none was conducted during a full season. Thus, future research should examine fitness and fatigue outcomes, internal and external training load data, and well-being questionnaires responses during a longer period (i.e., at least one full season) to better understand the relationship of different monitoring strategies in volleyball athletes. Besides, only five studies analysed fitness and fatigue in this athletic population [18, 28, 34, 42, 49]. Moreover, none of these studies was performed during a full season and future research should point in that direction. More specifically, fatigue in female volleyball athletes can be even more expanded by analysing the menstrual tracking and biochemical markers to develop a further understanding of how Ln rMSSD responses influence training adaptations.

Although the jump analysis is accepted as a reflection of external load, displacements and changes of direction also seem to affect this dimension (especially for the libero position). Therefore, those movements should be considered in future research as only one study analysed these metrics in a sample of collegiate female volleyball athletes [33]. Furthermore, the simple jump count method is not ideal to measure external load. Six studies expressed external load by analysing the jump height of each athlete [18, 27, 33, 39, 43, 46]. Still, two volleyball players with different body mass that achieve the same jumping height will not experience the same load. Due to gravity, linear velocity at landing increases with higher jumping height values, which subsequently increases kinetic energy (i.e., energy related to the body mass) levels at landing [83]. So, coaches should consider the vertical displacement of each jump as well as the mass of the athlete to have a better external load metric that is more reflective of what the volleyball athlete is experiencing [83]. Future research should explore the prospective relationship between external load calculated with the parameters mentioned before, the incidence of injury and the landing mechanics of volleyball players. This would potentially inform training and match-play guidelines by designing thresholds for injury prevention purposes.

One notable limitation in the current volleyball literature, and a promising direction for future research, is the exploration of GPS and Local Positioning Systems (LPS) for monitoring external load. While extensively used in outdoor sports, the application of GPS in volleyball, particularly indoor, is less common [84]. However, advancements in LPS technology now allow for its potential application in indoor environments, such as volleyball courts [85]. The adoption of these systems could provide detailed insights into player movements, intensity, and workload, which are crucial for training optimization, performance enhancement, and injury prevention [5, 85]. This area remains under-researched in volleyball, highlighting a significant gap and an opportunity for future studies. It is recommended that subsequent research investigates the utility and implementation of these technologies in volleyball, offering a comprehensive perspective on managing external load in athletes. Such exploration could substantially contribute to the evolving landscape of volleyball training and competition analysis.

The average CMJ height is more sensitive than highest CMJ height in monitoring the effects of fatigue [77]. However, three of the four studies that used this test to monitor neuromuscular fatigue opted to use the best of all attempts [28, 42, 49]. So, average CMJ height should be used in future volleyball studies to track neuromuscular status. Additionally, peak power, mean power, peak velocity, peak force, mean impulse, and calculated power would seem merit worthy in quantifying supercompensation effects [77] and no study evaluated the impact of these variables within volleyball athletes. Nevertheless, the more useful indicators of readiness and neuromuscular fatigue within the plethora of variables that the CMJ give are the FT:CT and reactive strength index modified (RSI_mod_) [86]. The RSI_mod_ is obtained by dividing the jump height to the contraction time and, similarly to FT:CT, the emphases of these two variables are jump process and force production [87]. Because time and contraction-specific measures better reflect the strategy employed by the neuromuscular system, compared with jumping height, contraction time is more sensitive to detect adaptations resulted from fatigue [88]. Since the ability of vertical jump height to reflect fatigue in athletes show inconsistencies in the literature [89, 90], future studies in volleyball should consider the use of RSI_mod_ and FT:CT to monitor neuromuscular fatigue.

While the CMJ test is prevalently used in the current literature, exploring alternative assessments could provide a more comprehensive understanding of neuromuscular responses in volleyball athletes. Tests like the Drop Jump, which involves a short-duration stretch–shortening cycle, can offer insights into reactive strength and plyometric capabilities under fatigued conditions [87]. Additionally, isometric tests, such as isometric mid-thigh pulls or isometric calf raises, could be utilized to assess force in specific joint positions [91]. These alternative tests could reveal different dimensions of fatigue that may not be fully captured by the CMJ alone. Incorporating a variety of neuromuscular assessments can help in developing a more nuanced understanding of fatigue patterns in volleyball players, which in turn could inform more effective training and recovery protocols. Therefore, it is recommended that future research in volleyball expand the repertoire of fatigue assessment tools to include dynamic, plyometric, and isometric evaluations, providing a broader spectrum of data to optimize athlete performance.

Finally, to mitigate divergency in fatigue, relative velocity loss thresholds have recently been implemented during the strength training prescription [92]. Thus, velocity based training (VBT) can be a great alternative to the most used percentage-based methods since the latter do not have into consideration training-related fatigue [93]. Therefore, strength and conditioning coaches should consider monitoring velocity attained at the start of a training session to help objectively monitor changes in athlete fitness and fatigue. This is a topic that needs more understanding and future research should seek to answer if VBT is a reliable and valid tool to monitor neuromuscular fatigue in volleyball athletes.

Due to the heterogeneity of the measures used, it was not possible to conduct a meta-analysis. Plus, RPE and well-being data can be collected without following specific procedures and across a range of methods (e.g., different RPE scales and/or different operational questions). Therefore, practitioners working in professional volleyball can use this information in various ways with different assessment standards between them and this systematic review did not have that into consideration. Nevertheless, since there is a growing interest in topics related to athletes’ monitoring this study can aid volleyball coaches to select which training load measures, fatigue and well-being assessments can be used with their athletes.

## Conclusions

Within the context of team sport athletes, such as volleyball, coaches should use a mixed-methods approach when monitoring these athletes. No single measure can determine how a player is fully coping with the demands of training and matches. Therefore, practitioners not only need a range of methods, but also ensure athletes are familiarized with them to better improve their buy-in and the quality of the data analysis. According to this review, internal training load should be collected daily after training sessions and matches with the sRPE method. External training load should also be measured daily according to the method proposed by Charlton et al. [83] based on jump height, jump count, and kinetic energy. If force platforms are available, neuromuscular fatigue can be assessed weekly using the FT:CT ratio of a CMJ or, in cases where force platforms are not available, the average jump height can also be used. Finally, the Hooper Index has been shown to be a measure of overall wellness, fatigue, stress, muscle soreness, mood, and sleep quality in volleyball when used daily.

### Supplementary Information

**Additional file1: Table S1.** PRISMA 2020 Checklist. **Table S2.** Definition of types of monitoring interventions. **Table S3.** Questions from the modified Downs and Black checklist used to evaluate methodological quality of the included articles. **Table S4.** Results of methodological quality assessment for included articles.

## Data Availability

All data are available upon request to the corresponding author.

## References

[1] Hopkins WG (1991). Quantification of training in competitive sports. Methods and applications Sports Med.

[2] Halson SL (2014). Monitoring Training Load to Understand Fatigue in Athletes. Sports Med.

[3] Veugelers KR, Young WB, Fahrner B, Harvey JT (2016). Different methods of training load quantification and their relationship to injury and illness in elite Australian football. J Sci Med Sport.

[4] Kellmann M (2010). Preventing overtraining in athletes in high-intensity sports and stress/recovery monitoring. Scand J Med Sci Sports.

[5] Rebelo A, Martinho DV, Valente-dos-Santos J, Coelho-e-Silva MJ, Teixeira DS (2023). From data to action: a scoping review of wearable technologies and biomechanical assessments informing injury prevention strategies in sport. BMC Sports Sci Med Rehabil.

[6] Gabbett TJ (2010). The development and application of an injury prediction model for noncontact, soft-tissue injuries in elite collision sport athletes. J Strength Cond Res.

[7] Svendsen IS, Gleeson M, Haugen TA, Tønnessen E (2015). Effect of an intense period of competition on race performance and self-reported illness in elite cross-country skiers. Scand J Med Sci Sports.

[8] Aubry A, Hausswirth C, Louis J, Coutts AJ (2014). Y LEM Functional overreaching: the key to peak performance during the taper?. Med Sci Sports Exerc..

[9] Nederhof E, Zwerver J, Brink M, Meeusen R, Lemmink K (2008). Different diagnostic tools in nonfunctional overreaching. Int J Sports Med.

[10] Meeusen R, Duclos M, Foster C, Fry A, Gleeson M, Nieman D (2013). Prevention, diagnosis, and treatment of the overtraining syndrome: joint consensus statement of the European College of Sport Science and the American College of Sports Medicine. Med Sci Sports Exerc.

[11] Issurin VB (2010). New horizons for the methodology and physiology of training periodization. Sports Med.

[12] Rebelo A, Pereira JR, Valente-dos-Santos J (2023). Effects of a preseason triphasic resistance training program on athletic performance in elite volleyball players—an observational study. German Journal of Exercise and Sport Research.

[13] Marques MC, Tillaar R, Vescovi JD, González-Badillo JJ (2008). Changes in strength and power performance in elite senior female professional volleyball players during the in-season: a case study. J Strength Cond Res.

[14] Smith DJ, Roberts D, Watson B (1992). Physical, physiological and performance differences between Canadian national team and universiade volleyball players. J Sports Sci.

[15] Puhl J, Case S, Fleck S, Van Handel P (1982). Physical and Physiological Characteristics of Elite Volleyball Players. Res Q Exerc Sport.

[16] Timoteo T, Seixas M, Falci M, Debien P, Miloski B, Miranda R (2017). Impact of consecutive games on workload, state of recovery and well-being of professional volleyball players. J Exerc Physiol Online.

[17] Timoteo T, Debien PB, Miloski B, Werneck FZ, Gabbett T, Bara Filho MG (2021). Influence of Workload and Recovery on Injuries in Elite Male Volleyball Players. J Strength Cond Res.

[18] Rebelo A, Pereira JR, Martinho DV, Amorim G, Lima R, Valente-Dos Santos J. Training Load, Neuromuscular Fatigue, and Well-Being of Elite Male Volleyball Athletes During an In-Season Mesocycle. Int J Sports Physiol Perform. 2023;18(4):354–62. 10.1123/ijspp.2022-0279 36754058

[19] Lima RF, Silva AF, Matos S, de Oliveira CH, Rebelo A, Clemente FM (2023). Using inertial measurement units for quantifying the most intense jumping movements occurring in professional male volleyball players. Sci Rep.

[20] Rebelo A, Pereira JR, Martinho DV, Valente-dos-Santos J. The Well-Being of Elite Volleyball Athletes: A Scoping Review of Methods Using Wellness Questionnaires. J Clin Sport Psychol. 2023;1–23.

[21] Reina M, García-Rubio J, Ibáñez SJ. Training and Competition Load in Female Basketball: A Systematic Review. Int J Environ Res Public Health. 2020;17(8):2639. 10.3390/ijerph17082639 PMC7215482 32290569

[22] Page MJ, McKenzie JE, Bossuyt PM, Boutron I, Hoffmann TC, Mulrow CD (2021). The PRISMA 2020 statement: an updated guideline for reporting systematic reviews. BMJ.

[23] Kohl C, McIntosh EJ, Unger S, Haddaway NR, Kecke S, Schiemann J (2018). Online tools supporting the conduct and reporting of systematic reviews and systematic maps: a case study on CADIMA and review of existing tools. Environ Evid.

[24] Downs SH, Black N (1998). The feasibility of creating a checklist for the assessment of the methodological quality both of randomised and non-randomised studies of health care interventions. J Epidemiol Community Health.

[25] Fox AS, Bonacci J, McLean SG, Spittle M, Saunders N (2014). What is normal? Female lower limb kinematic profiles during athletic tasks used to examine anterior cruciate ligament injury risk: a systematic review. Sports Med.

[26] Fox JL, Stanton R, Sargent C, Wintour SA, Scanlan AT (2018). The Association Between Training Load and Performance in Team Sports: A Systematic Review. Sports Med.

[27] Herring CH, Fukuda DH. Monitoring Competition Jump Load in Division I Female Collegiate Volleyball Athletes. J Sci Sport Exerc. 2022;53:43.

[28] Rabbani M, Agha-Alinejad H, Gharakhanlou R, Rabbani A, Flatt A. Monitoring training in women’s volleyball: Supine or seated heart rate variability? Physiol Behav. 2021. Ahead of print. 10.1016/j.physbeh.2021.113537 34331956

[29] Haraldsdottir K, Sanfilippo J, McKay L, Watson AM (2021). Decreased Sleep and Subjective Well-Being as Independent Predictors of Injury in Female Collegiate Volleyball Players. Orthop J Sports Med.

[30] Berriel GP, Peyré-Tartaruga LA, Lopes TR, Schons P, Zagatto AM, Sanchez-Sanchez J (2022). Relationship between vertical jumping ability and endurance capacity with internal training loads in professional volleyball players during preseason. J Sports Med Phys Fitness.

[31] Andrade DM, Fernandes G, Miranda R, Reis Coimbra D, Bara Filho MG (2021). Training Load and Recovery in Volleyball During a Competitive Season. J Strength Cond Res.

[32] Ungureanu AN, Brustio PR, Boccia G, Rainoldi A, Lupo C (2021). Effects of Presession Well-Being Perception on Internal Training Load in Female Volleyball Players. Int J Sports Physiol Perform.

[33] Kupperman N, Curtis MA, Saliba SA, Hertel J. Quantification of Workload and Wellness Measures in a Women’s Collegiate Volleyball Season. Front Sports Act Living. 2021;3:702419. 10.3389/fspor.2021.702419 PMC8377283 34423291

[34] Berriel GP, Costa RR, da Silva ES, Schons P, de Vargas GD, Peyré-Tartaruga LA (2020). Stress and recovery perception, creatine kinase levels, and performance parameters of male volleyball athletes in a preseason for a championship. Sports Med - Open.

[35] Horta T, Lima P, Matta G, Freitas J, Miloski B, Vianna J (2020). Training load impact on recovery status in professional volleyball athletes. Revista Brasileira de Medicina do Esporte.

[36] García-de-Alcaraz A, Ramírez-Campillo R, Rivera-Rodríguez M, Romero-Moraleda B (2020). Analysis of jump load during a volleyball season in terms of player role. J Sci Med Sport.

[37] Roy X, Caya O, Charron J, Comtois AS, Sercia P. Using global and differential ratings of perceived exertion to measure internal training load in university volleyball players. J Aust Strength Cond. 2020;28:6–13.

[38] Clemente FM, Silva AF, Clark CCT, Conte D, Ribeiro J, Mendes B (2020). Analyzing the Seasonal Changes and Relationships in Training Load and Wellness in Elite Volleyball Players. Int J Sports Physiol Perform.

[39] Lima RF, Silva A, Afonso J, Castro H, Clemente FM (2020). External and internal Load and their Effects on Professional Volleyball Training. Int J Sports Med.

[40] Duarte T, Reis Coimbra D, Miranda R, Toledo H, Werneck F, Freitas D (2019). Monitoring training load and recovery in volleyball players during a season. Revista Brasileira de Medicina do Esporte.

[41] Clemente FM, Mendes B, Palao JM, Silvério A, Carriço S, Calvete F (2019). Seasonal player wellness and its longitudinal association with internal training load: study in elite volleyball. J Sports Med Phys Fitness.

[42] Horta T, Bara Filho MG, Coimbra DR, Miranda R, Werneck FZ (2019). Training Load, Physical Performance, Biochemical Markers, and Psychological Stress During a Short Preparatory Period in Brazilian Elite Male Volleyball Players. J Strength Cond Res.

[43] Silva  D, Vázquez J, Ramos J, Clemente FM, Camões M, Lima RF (2019). Intra-week variations and associations between internal and external load measures in a elite volleyball team. J Hum Sport & Exerc.

[44] Roy X, Comtois AS, Sercia P. Relationship between daily training loads and perceptions of wellness in Canadian university volleyball athletes. J Aust Strength Cond. 2019;27(7).

[45] Hyatt HW, Kavazis AN (2019). Body Composition and Perceived Stress through a Calendar Year in NCAA I Female Volleyball Players. Int J Exerc Sci.

[46] Skazalski C, Whiteley R, Bahr R (2018). High jump demands in professional volleyball-large variability exists between players and player positions. Scand J Med Sci Sports.

[47] Castello M, Reed J, Lund R. Relationship Between Physical Training, Ratings of Perceived Exertion, and Mental Toughness in Female NCAA Division I Volleyball Players. Sport J. 2018;21:1–8.

[48] Mendes B, Palao JM, Silvério A, Owen A, Carriço S, Calvete F (2018). Daily and weekly training load and wellness status in preparatory, regular and congested weeks: a season-long study in elite volleyball players. Res Sports Med.

[49] Tavares F, Simões M, Matos B, Smith TB, Driller M (2018). Wellness, muscle soreness and neuromuscular performance during a training week in volleyball athletes. J Sports Med Phys Fitness.

[50] Debien PB, Mancini M, Coimbra DR, de Freitas DGS, Miranda R, Bara Filho MG (2018). Monitoring Training Load, Recovery, and Performance of Brazilian Professional Volleyball Players During a Season. Int J Sports Physiol Perform.

[51] Brandão FM, Cunha VF, Timoteo TF, Duarte TS, Dias BM, Coimbra DR, et al. Behavior of the training load, recovery and well-being in volleyball professional athletes in weeks with and without matches. Educación física y ciencia. 2018;20(4).

[52] Nogueira F, Miloski B, Filho M, Lourenço L (2017). Influence of the presence or absence of games in athletes volleyball professionals fatigue perceptions during a competitive season. Revista Portuguesa de Ciências do Desporto.

[53] Horta T, Reis Coimbra D, Miranda R, Werneck F, Filho M (2017). Is the internal training load different between starters and nonstarters volleyball playerssubmitted to the same external load training? A case study. Revista Brasileira de Cineantropometria e Desempenho Humano.

[54] de Freitas V, Nakamura F, Nogueira F, Pereira L, Reis Coimbra D, Filho M. Pre-competitive physical training and markers of performance, stress and recovery in young volleyball athletes. Revista Brasileira de Cineantropometria e Desempenho Humano. 2015;17(1).

[55] Impellizzeri FM, Rampinini E, Marcora SM (2005). Physiological assessment of aerobic training in soccer. J Sports Sci.

[56] Fowles JR (2006). Technical issues in quantifying low-frequency fatigue in athletes. Int J Sports Physiol Perform.

[57] Cormack SJ, Mooney MG, Morgan W, McGuigan MR (2013). Influence of neuromuscular fatigue on accelerometer load in elite Australian football players. Int J Sports Physiol Perform.

[58] Gathercole R, Sporer B, Stellingwerff T, Sleivert G (2015). Alternative countermovement-jump analysis to quantify acute neuromuscular fatigue. Int J Sports Physiol Perform.

[59] Taylor K-L, Chapman D, Cronin J, Newton M, Gill N (2012). Fatigue Monitoring in High Performance Sport: A Survey of Current Trends. Journal of Australian Strength and Conditioning.

[60] Plews DJ, Laursen PB, Kilding AE, Buchheit M (2013). Evaluating training adaptation with heart-rate measures: a methodological comparison. Int J Sports Physiol Perform.

[61] McLean BD, Coutts AJ, Kelly V, McGuigan MR, Cormack SJ (2010). Neuromuscular, endocrine, and perceptual fatigue responses during different length between-match microcycles in professional rugby league players. Int J Sports Physiol Perform.

[62] Urhausen A, Gabriel H, Kindermann W (1995). Blood hormones as markers of training stress and overtraining. Sports Med.

[63] Saw AE, Main LC, Gastin PB (2016). Monitoring the athlete training response: subjective self-reported measures trump commonly used objective measures: a systematic review. Br J Sports Med.

[64] Lane AM, Terry PC, Stevens MJ, Barney S, Dinsdale SL (2004). Mood responses to athletic performance in extreme environments. J Sports Sci..

[65] Hooper SL, Mackinnon LT, Howard A, Gordon RD, Bachmann AW (1995). Markers for monitoring overtraining and recovery. Med Sci Sports Exerc.

[66] Miloski B, de Freitas VH, Nakamura FY (2016). de ANFC, Bara-Filho MG Seasonal Training Load Distribution of Professional Futsal Players Effects on Physical Fitness, Muscle Damage and Hormonal Status. J Strength Cond Res.

[67] Chiu LZF, Barnes JL (2003). The Fitness-Fatigue Model Revisited: Implications for Planning Short- and Long-Term Training. Strength & Conditioning Journal.

[68] Gabbett TJ, Jenkins DG (2011). Relationship between training load and injury in professional rugby league players. J Sci Med Sport.

[69] Araújo R, Mesquita I, Marcelino R (2009). Relationship between Block Constraints and set outcome in Elite Male Volleyball. Int J Perform Anal Sport.

[70] Impellizzeri FM, Rampinini E, Coutts AJ, Sassi A, Marcora SM (2004). Use of RPE-Based Training Load in Soccer. Med Sci Sports Exerc.

[71] Buchheit M (2014). Monitoring training status with HR measures: do all roads lead to Rome?. Front Physiol.

[72] Flatt AA, Howells D (2019). Effects of varying training load on heart rate variability and running performance among an Olympic rugby sevens team. J Sci Med Sport.

[73] Plews DJ, Laursen PB, Kilding AE, Buchheit M (2012). Heart rate variability in elite triathletes, is variation in variability the key to effective training? A case comparison. Eur J Appl Physiol.

[74] Mourot L, Bouhaddi M, Tordi N, Rouillon JD, Regnard J (2004). Short- and long-term effects of a single bout of exercise on heart rate variability: Comparison between constant and interval training exercises. Eur J Appl Physiol.

[75] Brancaccio P, Maffulli N, Limongelli FM (2007). Creatine kinase monitoring in sport medicine. Br Med Bull.

[76] Guilhem G, Hanon C, Gendreau N, Bonneau D, Guével A, Chennaoui M (2015). Salivary Hormones Response to Preparation and Pre-competitive Training of World-class Level Athletes. Front Physiol.

[77] Claudino JG, Cronin J, Mezêncio B, McMaster DT, McGuigan M, Tricoli V (2017). The countermovement jump to monitor neuromuscular status: A meta-analysis. J Sci Med Sport.

[78] Watson A, Johnson M, Sanfilippo J (2020). Decreased Sleep Is an Independent Predictor of In-Season Injury in Male Collegiate Basketball Players. Orthopaedic journal of sports medicine..

[79] Leeder J, Glaister M, Pizzoferro K, Dawson J, Pedlar C (2012). Sleep duration and quality in elite athletes measured using wristwatch actigraphy. J Sports Sci.

[80] Simpson NS, Gibbs EL, Matheson GO (2017). Optimizing sleep to maximize performance: implications and recommendations for elite athletes. Scand J Med Sci Sports.

[81] Keaney LC, Kilding AE, Merien F, Dulson DK (2018). The impact of sport related stressors on immunity and illness risk in team-sport athletes. J Sci Med Sport.

[82] Madrigal LA, Wilson PB (2017). Salivary Hormone and Anxiety Responses to Free-Throw Shooting Competition in Collegiate Female Basketball Players. J Clin Sport Psychol.

[83] Charlton PC, Kenneally-Dabrowski C, Sheppard J, Spratford W (2017). A simple method for quantifying jump loads in volleyball athletes. J Sci Med Sport.

[84] Cummins C, Orr R, O’Connor H, West C (2013). Global Positioning Systems (GPS) and Microtechnology Sensors in Team Sports: A Systematic Review. Sports Med.

[85] Conte D (2020). Validity of local positioning systems to measure external load in sport settings: a brief review. Hum Mov.

[86] Martinez D (2006). From the field-directed topic: The use of reactive strength index, reactive strength infex modified, and flight time: Contraction time as monitoring tools. J Aust Strength Cond.

[87] Rebelo A, Pereira JR, Martinho DV, Duarte JP, Coelho-e-Silva MJ, Valente-dos-Santos J (2022). How to Improve the Reactive Strength Index among Male Athletes? A Systematic Review with Meta-Analysis. Healthcare.

[88] Cormack SJ, Newton RU, McGuigan MR (2008). Neuromuscular and endocrine responses of elite players to an Australian rules football match. Int J Sports Physiol Perform.

[89] Ronglan LT, Raastad T, Børgesen A (2006). Neuromuscular fatigue and recovery in elite female handball players. Scand J Med Sci Sports.

[90] Coutts AJ, Reaburn P, Piva TJ, Rowsell GJ (2007). Monitoring for overreaching in rugby league players. Eur J Appl Physiol.

[91] Comfort P, Dos'Santos T, Beckham GK, Stone MH, Guppy SN, Haff GG (2019). Standardization and Methodological Considerations for the Isometric Midthigh Pull. Strength & Conditioning Journal.

[92] Weakley J, Ramirez-Lopez C, McLaren S, Dalton-Barron N, Weaving D, Jones B (2020). The Effects of 10%, 20%, and 30% Velocity Loss Thresholds on Kinetic, Kinematic, and Repetition Characteristics During the Barbell Back Squat. Int J Sports Physiol Perform.

[93] Weakley J, Mann B, Banyard H, McLaren S, Scott T, Garcia-Ramos A (2021). Velocity-Based Training: From Theory to Application. Strength & Conditioning Journal.

