# Supplementary material for: Training stress, neuromuscular fatigue and well-being in volleyball: a systematic review
Source: BMC Sports Sci Med Rehabil. 2024 Jan 13;16:17. doi: 10.1186/s13102-024-00807-7 (PMC10788005; doi:10.1186/s13102-024-00807-7)
Supplement: Supplementary file 1 — Additional file1: Table S1. PRISMA 2020 Checklist. Table S2. Definition of types of monitoring interventions. Table S3. Questions from the modified Downs and Black checklist used to evaluate methodological quality of the included articles. Table S4. Results of methodological quality assessment for included articles. [file 13102_2024_807_MOESM1_ESM.docx]

**SUPPLEMENTARY FILE**

**CONTENTS**

Table S1 PRISMA 2020 Checklist.

Table S2 Definition of types of monitoring interventions.

Table S3 Questions from the modified Downs and Black checklist used to evaluate methodological quality of the included articles.

Table S4 Results of methodological quality assessment for included articles.

**Table S1.** PRISMA 2020 Checklist.

| **Section and Topic** | **Item #** | **Checklist item** | **Location where item is reported** |
| --- | --- | --- | --- |
| **TITLE** | | |  |
| Title | 1 | Identify the report as a systematic review. | Page 1 |
| **ABSTRACT** | | |  |
| Abstract | 2 | See the PRISMA 2020 for Abstracts checklist. | Page 2 |
| **INTRODUCTION** | | |  |
| Rationale | 3 | Describe the rationale for the review in the context of existing knowledge. | Pages 3-4 |
| Objectives | 4 | Provide an explicit statement of the objective(s) or question(s) the review addresses. | Page 4 |
| **METHODS** | | |  |
| Eligibility criteria | 5 | Specify the inclusion and exclusion criteria for the review and how studies were grouped for the syntheses. | Page 5 |
| Information sources | 6 | Specify all databases, registers, websites, organisations, reference lists and other sources searched or consulted to identify studies. Specify the date when each source was last searched or consulted. | Page 5 |
| Search strategy | 7 | Present the full search strategies for all databases, registers and websites, including any filters and limits used. | Pages 5-6 |
| Selection process | 8 | Specify the methods used to decide whether a study met the inclusion criteria of the review, including how many reviewers screened each record and each report retrieved, whether they worked independently, and if applicable, details of automation tools used in the process. | Page 6 |
| Data collection process | 9 | Specify the methods used to collect data from reports, including how many reviewers collected data from each report, whether they worked independently, any processes for obtaining or confirming data from study investigators, and if applicable, details of automation tools used in the process. | Page 6 |
| Data items | 10a | List and define all outcomes for which data were sought. Specify whether all results that were compatible with each outcome domain in each study were sought (e.g. for all measures, time points, analyses), and if not, the methods used to decide which results to collect. | Pages 6-7 |
|  | 10b | List and define all other variables for which data were sought (e.g. participant and intervention characteristics, funding sources). Describe any assumptions made about any missing or unclear information. | Pages 6-7 |
| Study risk of bias assessment | 11 | Specify the methods used to assess risk of bias in the included studies, including details of the tool(s) used, how many reviewers assessed each study and whether they worked independently, and if applicable, details of automation tools used in the process. | Page 7 |
| Effect measures | 12 | Specify for each outcome the effect measure(s) (e.g. risk ratio, mean difference) used in the synthesis or presentation of results. | Page 7 |
| Synthesis methods | 13a | Describe the processes used to decide which studies were eligible for each synthesis (e.g. tabulating the study intervention characteristics and comparing against the planned groups for each synthesis (item #5)). | Page 7 |
|  | 13b | Describe any methods required to prepare the data for presentation or synthesis, such as handling of missing summary statistics, or data conversions. | Page 7 |
|  | 13c | Describe any methods used to tabulate or visually display results of individual studies and syntheses. | Page 7 |
|  | 13d | Describe any methods used to synthesize results and provide a rationale for the choice(s). If meta-analysis was performed, describe the model(s), method(s) to identify the presence and extent of statistical heterogeneity, and software package(s) used. | Page 7 |
|  | 13e | Describe any methods used to explore possible causes of heterogeneity among study results (e.g. subgroup analysis, meta-regression). | N/A |
|  | 13f | Describe any sensitivity analyses conducted to assess robustness of the synthesized results. | N/A |
| Reporting bias assessment | 14 | Describe any methods used to assess risk of bias due to missing results in a synthesis (arising from reporting biases). | N/A |
| Certainty assessment | 15 | Describe any methods used to assess certainty (or confidence) in the body of evidence for an outcome. | N/A |
| **RESULTS** | | |  |
| Study selection | 16a | Describe the results of the search and selection process, from the number of records identified in the search to the number of studies included in the review, ideally using a flow diagram. | Page 8 |
|  | 16b | Cite studies that might appear to meet the inclusion criteria, but which were excluded, and explain why they were excluded. | Page 8 |
| Study characteristics | 17 | Cite each included study and present its characteristics. | Pages 8-10 |
| Risk of bias in studies | 18 | Present assessments of risk of bias for each included study. | Page 8 |
| Results of individual studies | 19 | For all outcomes, present, for each study: (a) summary statistics for each group (where appropriate) and (b) an effect estimate and its precision (e.g. confidence/credible interval), ideally using structured tables or plots. | Pages 10-16 |
| Results of syntheses | 20a | For each synthesis, briefly summarise the characteristics and risk of bias among contributing studies. | Pages 10-16 |
|  | 20b | Present results of all statistical syntheses conducted. If meta-analysis was done, present for each the summary estimate and its precision (e.g. confidence/credible interval) and measures of statistical heterogeneity. If comparing groups, describe the direction of the effect. | Pages 10-16 |
|  | 20c | Present results of all investigations of possible causes of heterogeneity among study results. | Pages 10-16 |
|  | 20d | Present results of all sensitivity analyses conducted to assess the robustness of the synthesized results. | Pages 10-16 |
| Reporting biases | 21 | Present assessments of risk of bias due to missing results (arising from reporting biases) for each synthesis assessed. | N/A |
| Certainty of evidence | 22 | Present assessments of certainty (or confidence) in the body of evidence for each outcome assessed. | N/A |
| **DISCUSSION** | | |  |
| Discussion | 23a | Provide a general interpretation of the results in the context of other evidence. | Pages 16-20 |
|  | 23b | Discuss any limitations of the evidence included in the review. | Pages 21-23 |
|  | 23c | Discuss any limitations of the review processes used. | Pages 21-23 |
|  | 23d | Discuss implications of the results for practice, policy, and future research. | Pages 21-23 |
| **OTHER INFORMATION** | | |  |
| Registration and protocol | 24a | Provide registration information for the review, including register name and registration number, or state that the review was not registered. | Page 4 |
|  | 24b | Indicate where the review protocol can be accessed, or state that a protocol was not prepared. | Page 4 |
|  | 24c | Describe and explain any amendments to information provided at registration or in the protocol. | Page 4 |
| Support | 25 | Describe sources of financial or non-financial support for the review, and the role of the funders or sponsors in the review. | Page 24 |
| Competing interests | 26 | Declare any competing interests of review authors. | Page 24 |
| Availability of data, code and other materials | 27 | Report which of the following are publicly available and where they can be found: template data collection forms; data extracted from included studies; data used for all analyses; analytic code; any other materials used in the review. | Page 24 |

*From:*  Page MJ, McKenzie JE, Bossuyt PM, Boutron I, Hoffmann TC, Mulrow CD, et al. The PRISMA 2020 statement: an updated guideline for reporting systematic reviews. BMJ 2021;372:n71. doi: 10.1136/bmj.n71

**Table S2. Definition of types of monitoring interventions.**

| **Monitoring intervention** | **Definition** |
| --- | --- |
| Training stress | Internal training load: physiological stress that a training session induces in the athlete. Common measures include heart rate and rating of perceived exertion.  External training load: physical work prescribed in the training plan. The most common method is with time-motion analysis devices, such as GPS, accelerometers, or inertial motion units. |
| Fitness and fatigue | Low-frequency fatigue: resulted from high-force, high-intensity, or repeated stretch-shortening cycles muscle actions. Vertical jumps are the most used tests to assess neuromuscular fatigue in athletes. Submaximal exercise protocols and physiological markers such as heart rate can also be used as objective markers of fatigue. |
| Well-being | Wellness inventories and well-being questionnaires are the most common measures to assess metrics such as mood, stress, sleep quality, and muscle soreness in athletes. |

**Table S3. Questions from the modified Downs and Black checklist used to evaluate methodological quality of the included articles.**

| Question no. | Question |
| --- | --- |
|  | **Reporting** |
| 1 | Is the hypothesis/aim/objective of the study clearly described? |
| 2 | Are the main outcomes to be measured clearly described in the introduction or methods section? |
| 3 | Are the characteristics of the subjects included in the study clearly described? |
| 4 | Are the main findings of the study clearly described? |
| 5 | Does the study provide estimates of the random variability in the data for the main outcomes? |
| 6 | Have actual probability values been reported (e.g., 0.035 rather than < 0.05) for the main outcomes except where the probability value is < 0.001? |
|  | **External validity** |
| 7 | Were the subjects asked to participate in the study representative of the entire population from which they were recruited? |
| 8 | Were those subjects who were prepared to participate representative of the entire population from which they were recruited? |
|  | **Internal validity bias** |
| 9 | If any of the results of the study were based on “data dredging,” was this made clear? |
| 10 | Were the statistical tests used to assess the main outcomes appropriate? |
| 11 | Were the main outcome measures accurate (valid and reliable)? |

**Table S4. Results of methodological quality assessment for included articles.**

| Study | Downs and Black checklist question number | | | | | | | | | | | | | Total | |  |
| --- | --- | --- | --- | --- | --- | --- | --- | --- | --- | --- | --- | --- | --- | --- | --- | --- |
|  | Reporting | | | | | | External validity | | | Internal validity  bias | | | |  |  |  |
|  | 1 | 2 | 3 | 4 | 5 | 6 | | 7 | 8 | | 9 | 10 | 11 | |  | |
| Rebelo *et al*, 2023 [18] | 1 | 1 | 1 | 1 | 1 | 1 | | 0 | 0 | | 1 | 1 | 1 | | **9** | |
| Herring and Fukuda, 2022 [27] | 1 | 1 | 1 | 1 | 1 | 1 | | 0 | 0 | | 1 | 1 | 1 | | **9** | |
| Berriel *et al*, 2022 [30] | 1 | 1 | 1 | 1 | 1 | 1 | | 0 | 0 | | 1 | 1 | 1 | | **9** | |
| Rabbani *et al*, 2021 [28] | 1 | 1 | 1 | 1 | 1 | 1 | | 0 | 0 | | 1 | 1 | 1 | | **9** | |
| Haraldsdottir *et al*, 2021 [29] | 1 | 1 | 1 | 1 | 1 | 1 | | 0 | 0 | | 1 | 1 | 1 | | **9** | |
| Andrade *et al*, 2021 [31] | 1 | 1 | 1 | 1 | 1 | 0 | | 0 | 0 | | 1 | 1 | 1 | | **8** | |
| Timoteo *et al*, 2021 [17] | 1 | 1 | 1 | 1 | 1 | 1 | | 0 | 0 | | 1 | 1 | 1 | | **9** | |
| Ungureanu *et al*, 2021 [32] | 1 | 1 | 1 | 1 | 0 | 1 | | 0 | 0 | | 1 | 1 | 1 | | **8** | |
| Kupperman *et al*, 2021 [33] | 1 | 1 | 1 | 1 | 1 | 0 | | 0 | 0 | | 1 | 1 | 1 | | **8** | |
| Berriel *et al*, 2020 [34] | 1 | 1 | 1 | 1 | 1 | 1 | | 0 | 0 | | 1 | 1 | 1 | | **9** | |
| Horta *et al*, 2020 [35] | 1 | 1 | 1 | 0 | 1 | 1 | | 0 | 0 | | 1 | 1 | 1 | | **9** | |
| García-de-Alcaraz *et al*, 2020 [36] | 1 | 1 | 1 | 1 | 0 | 1 | | 0 | 0 | | 1 | 1 | 1 | | **8** | |
| Roy *et al*, 2020 [37] | 1 | 1 | 1 | 1 | 1 | 0 | | 0 | 0 | | 1 | 1 | 1 | | **8** | |
| Clemente *et al*, 2020 [38] | 1 | 1 | 1 | 1 | 1 | 1 | | 0 | 0 | | 1 | 1 | 1 | | **9** | |
| Lima *et al*, 2020 [39] | 1 | 1 | 1 | 1 | 0 | 1 | | 0 | 0 | | 1 | 1 | 1 | | **8** | |
| Duarte *et al*, 2019 [40] | 1 | 1 | 1 | 1 | 1 | 1 | | 0 | 0 | | 1 | 1 | 1 | | **9** | |
| Clemente *et al*, 2019 [41] | 1 | 1 | 1 | 1 | 0 | 1 | | 0 | 0 | | 1 | 1 | 1 | | **8** | |
| Horta *et al*, 2019 [42] | 1 | 1 | 1 | 1 | 0 | 1 | | 0 | 0 | | 1 | 1 | 1 | | **8** | |
| Silva *et al*, 2019 [43] | 1 | 1 | 1 | 1 | 1 | 0 | | 0 | 0 | | 1 | 1 | 1 | | **8** | |
| Roy *et al*, 2019 [44] | 1 | 1 | 1 | 1 | 1 | 0 | | 0 | 0 | | 1 | 1 | 1 | | **8** | |
| Hyatt and Kavazis, 2019 [45] | 1 | 1 | 1 | 1 | 1 | 0 | | 0 | 0 | | 1 | 1 | 1 | | **8** | |
| Skazalski *et al*, 2018 [46] | 1 | 1 | 1 | 1 | 0 | 0 | | 0 | 0 | | 1 | 1 | 1 | | **7** | |
| Castello *et al*, 2018 [47] | 1 | 1 | 1 | 1 | 0 | 1 | | 0 | 0 | | 1 | 1 | 1 | | **8** | |
| Mendes *et al*, 2018 [48] | 1 | 1 | 1 | 1 | 0 | 1 | | 0 | 0 | | 1 | 1 | 1 | | **8** | |
| Tavares *et al*, 2018 [49] | 1 | 1 | 1 | 1 | 0 | 1 | | 0 | 0 | | 1 | 1 | 1 | | **8** | |
| Debien *et al*, 2018 [50] | 1 | 1 | 1 | 1 | 1 | 0 | | 0 | 0 | | 1 | 1 | 1 | | **8** | |
| Brandão *et al*, 2018 [51] | 1 | 1 | 1 | 0 | 0 | 1 | | 0 | 0 | | 1 | 1 | 1 | | **6** | |
| Nogueira *et al*, 2017 [52] | 1 | 1 | 1 | 1 | 1 | 0 | | 0 | 0 | | 1 | 1 | 1 | | **8** | |
| Horta *et al*, 2017 [53] | 1 | 1 | 1 | 1 | 0 | 1 | | 0 | 0 | | 1 | 1 | 1 | | **8** | |
| Timoteo *et al*, 2017 [16] | 1 | 1 | 1 | 1 | 0 | 1 | | 0 | 0 | | 1 | 1 | 1 | | **8** | |
| de Freitas *et al*, 2015 [54] | 1 | 1 | 1 | 1 | 1 | 0 | | 0 | 0 | | 1 | 1 | 1 | | **8** | |

1 = yes; 0 = no/unable to determine.
